# Conference Report: COMPASS ’94, NINTH ANNUAL CONFERENCE ON COMPUTER ASSURANCE Gaithersburg, MD June 27–July 1, 1994

**DOI:** 10.6028/jres.099.072

**Published:** 1994

**Authors:** Laura M. Ippolito, Dolores R. Wallace, Elizabeth B. Lennon

**Affiliations:** Computer Systems Laboratory, National Institute of Standards and Technology, Gaithersburg, MD 20899-0001

## 1. Introduction

Cosponsored by the IEEE Aerospace and Electronics Systems Society and the IEEE National Capital Area Council, COMPASS is an organization which advances the theory and practice of building computer assurance into critical systems. NIST’s Computer Systems Laboratory hosted the Ninth Annual Conference on Computer Assurance (COMPASS ’94) on June 27-July 1, 1994, and served as cosponsors with the following industry and government organizations: Area Systems, Inc.; Booz-Allen & Hamilton; CSA (Control Systems Analysis, Inc.); Kaman Sciences Corporation; Logicon, Inc.; National Institute of Standards and Technology; Naval Research Laboratory; Naval Surface Warfare Center; Systems Safety Society; Trusted Information Systems; TRW Systems Division; and the U.S. General Accounting Office. COMPASS ’94 attracted more than 150 participants from government, industry, academia, and foreign countries such as Canada, England, Japan, Germany, Scotland, Korea, and Sweden. This year’s focus was on the use and assessment of formal methods and on alternatives to formal verification in the critical areas of safety, reliability, fault tolerance, concurrency and real time, and security.

## 2. Tutorials

COMPASS ’94 featured two full-day tutorials and two half-day tutorials. In the first full-day tutorial, John McDermid (University of York) and Christopher Locke (York Software Engineering Limited) discussed “Formal Software Development Using Z.” The general characteristics of formal methods for software development were addressed, examples of using the Z Formal Method were given, and a demonstration of the tool CADIZ (Computer Aided Design in Z) was performed.

Hans-Ludwig Hausen (German National Research Center for Computer Science) gave the second full-day tutorial on “Software System Evaluation and Certification.” This tutorial focused on the methods and tools for the evaluation and assessment of software products and processes. Particular emphasis was given to identifying and selecting software characteristics and metrics and the handling of evaluation methods and tools.

The first half-day tutorial on “Software Hazard Analysis” was given by Nancy Leveson (University of Washington). This tutorial presented information on techniques used to construct safe and correct process-control software. A system engineering approach was described, where the software hazard analysis is conducted to ensure requirements specifications are consistent with system safety constraints. Some examples of the formal techniques used on the Traffic Alert and Collision Avoidance System (TCAS) II project were also provided.

Marvin Schaefer (Area Systems, Inc.) conducted the final, half-day tutorial on “Trusted DBMS Considerations and Issues.” He addressed issues connected with building trusted DBMS’s, and presented current state of the art and trends. Major risks and problems were also discussed.

## 3. General Conference

The first full day of the conference opened with welcoming remarks by H. O. Lubbes and Jan Filsinger, COMPASS ’94 General Co-Chairs, and John McLean, COMPASS ’94 Program Chair. This was the first year that COMPASS included a tools fair. Nine vendors exhibited fifteen tools: Risk Watch (Expert Systems Software, Inc.); AeSOP and Aries (The Aerospace Corporation); EVES (ORA–Ottawa); AdaWise, Penelope, Romulus, and Larch-Ada (ORA–Ithaca), McCabe Toolset (McCabe & Associates); ModeChart Toolset (NRL); Centurion (SRS Technologies); RDD-100 (Ascent Logic Corporation); Boundary Flow Covert Channel Analysis (CTA, Inc.); INTERLOCKS (CSA); and FDR Tool (formal systems ltd).

Jerry O. Tuttle, VADM USN (RET.), delivered the keynote address on the importance of computer systems in the present-day world. The dependence on critical systems demands that the systems are built with safety and security assurances. Tuttle noted the explosion in information and ever-increasing need to build secure systems not only in military systems but also in industry. He noted that “opportunity is often disguised as unsolvable problems.” He noted that this challenge to improve technology to make systems safe and secure should be accepted.

## 4. Safety I

The first paper of the conference, “Experience Applying the CoRE Method to the Lockheed C-130J Software Requirements,” was presented by Stuart Faulk, Lisa Finneran, and James Kirby (SPC), and James Sutton (Time Plus). It described the CoRE class model, a descendant of the Ward/Mellor Structured Analysis method, and its application to the C130J project.

Stephen S. Cha (The Aerospace Corporation) discussed “AeSOP: An Interactive Failure Mode Analysis Tool.” His presentation included a demonstration of the AeSOP tool to assist in fault tree analysis using petri-nets.

“A Development of Hazard Analysis to Aid Software Design” was presented by John McDermid and D. J. Pumfrey (University of York). This talk described the application of the technique of HAZOP (Hazard and Operability Studies), adapted from the chemical industry, to hazard analysis.

## 5. Use and Assessment of Formal Methods

David Guaspari (ORA) began this session with a paper on “Formal Methods in the Design of Ada 9x.” He related experiences of using a mathematical model for verifying the design of the Language Precision Team that is revising Ada 9x language.

A “Case Study: Applying Formal Methods to the Traffic Alert and Collision Avoidance System (TCAS) 11” was detailed by Joan J. Britt (MITRE). She described the TCAS II System Requirements Specification written in RSML (Requirements State Machine Language), illustrating how formal methods have been applied to this safety critical system. Britt noted improvements in qualify assurance in three areas: product review, process and personnel certification, and functional testing. She also proposed improvements that can develop RSML into a methodology.

“Formal Methods and Dependability Assessment” was presented by V. Stavridou, S. Liu, and B. Dutertre (University of London). The fact that formal methods are used increasingly for system development was discussed. Their potential advantages for dependability assurance have been recognized. However, no measurable evidence exists that supports or refutes the efficacy of formal methods.

## 6. Alternatives to Formal Verincation

This session featured two papers. “Using Formal Methods To Derive Test Frames In Category-Partition Testing” was presented by Paul Ammann and Jeff Offutt (George Mason University). “Application Of An Informal Program Verification Method To Ada” was presented by Bruce Wieand (IBM) and William E. Howden (University of California). Both presentations dealt with verification; however, they covered activities that apply to different stages in the software life-cycle. The first paper discussed mechanization of requirements test suite derivation while the second proposed enhancements to code inspection process.

Offutt presented an extension of category partitioning, a specification-based testing method to mechanize construction of test specifications. An application of this method was shown using an example study of a simple file system. The authors believe that this formalization of the notion of a test specification fills the large gap between the functional specifications and the actual test cases. Further, this formality allows mechanization of test specifications so that the tester can focus on only the aspects of testing that demand engineering judgment. The method can be employed early in the life-cycle of the project, and the products from this step (coverage metric and the test specification) are useful in determining when to stop testing. The experimenters concluded that the method is relatively inexpensive and feasible.

Wieand presented the QDA (Quick Defect Analysis) informal program verification method as an aid to code inspection. Previous work in QDA has shown this method to be effective for assembly language programs. The current prototype is an application of QDA to Ada. The method essentially verifies all assumptions by associating objects and their properties. Any unconfirmed hypothesis triggers an investigation probably leading to a program fault or an error in assumption. This experiment has proved that the method (with appropriate enhancements) is applicable to a high-level language.

## 7. Fault Tolerance

“Centurion Software Fault Tolerance Design and Analysis Tool” by G. Steve Wakefield (SRS), Roger Dziegiel (USAF Rome Laboratory), and Laura L. Pullum (Quality Research Associates) described Centurion, a computer-aided software fault tolerance design and analysis tool. This tool may be used to evaluate software and the associated computer and communications hardware.

Cristian Constantinescu (Duke University) presented “Estimation of Coverage Probabilities for Dependability Validation of Fault-Tolerant Computing System.” Coverage probability is estimated by statistically processing information collected through physical or simulated fault injection. The statistical experiments are carried out in a three-dimensional fault space that accounts for system inputs, fault injection times, and fault locations. The proposed solution technique is tested against the data generated by a program that mimics a fault environment.

“Formal Verification of an Interactive Consistency Algorithm for the Draper FTP Architecture Under a Hybrid Fault Model” is the subject of a paper by Patrick Lincoln and John Rushby (SRI International). A hybrid fault model as opposed to the classical Byzantine model was presented to be used on an asymmetric architecture. Although this scheme reduces the number of processors needed to withstand a given number of faults, this extended fault model and the asymmetric architecture complicate the arguments for correctness.

## 8. Concurrency and Real-Time Systems

Inhye Kang and Insup Lee (University of Pennsylvania) presented a paper on “State Minimization for Concurrent System Analysis Based on State Space Exploration.” They discussed a method to compress similar states in the reachable state space during concurrent system analysis.

“Compositional Model Checking of Ada Tasking Programs” by Jeffrey Fischer (Verdix) and Richard Gerber (University of Maryland) discussed another method of state space compression by analyzing a subsection of the state space first and reducing it to a smaller graph.

Azer Bestavros (Boston University) presented “An Ounce of Prevention is Worth a Pound of Cure: Towards Physically-Correct Specifications of Embedded Real-Time Systems.” This presentation covered CLEOPATRA, a methodology that prevents system specification that have certain physically impossible specifications (timing, infinite capacity, etc.).

## 9. Panel: Software Testability for Critical Systems

The four members of the panel were Jeff Voas (Reliable Software Technologies Corporation), Dick Hamlet (Portland State University), William E. Howden (University of California at La Jolla), and Keith Miller (Sangamon State University). Jeff Voas talked about testability, testing and critical software assessment. Complexity measures and coverage criteria are only two classes of measures in the class of testability metrics. Software testability is a metric that analyzes the code itself in a “white-box” fashion. Testability measurement plan will decrease development costs and will not in any way slow down progress.

In Dick Hamlet’s absence, Keith Miller presented Hamlet’s views on software reliability. He discussed software reliability that is inherently dependent on the very nature of software. One cannot measure software reliability with efforts made in development. Instead, we ought to seek the relationship between defect-detection methods employed during the development and the quality of these methods. W. E. Howden’s presented views on testability, failure rates, detectability, trustability and reliability.

Keith W. Miller discussed testability, including its theoretical aspects, its practical implementation, and its application to reliability estimation. He described the complementary advantages and disadvantages of random testing and testability analysis. Finally, he explained how a fully automated system, such as PISCES, can make testability analysis possible without any oracle for correctness.

## 10. Hardware Verification

The first paper of the session was “A Formal Model of Several Fundamental VHDL Concepts” by David M. Goldschlag (NRL). This presentation began with a brief introduction to VHDL. The key concepts of VHDL, concurrency, real time, and event driven simulation, were discussed and Goldschlag proposed an extension to VHDL: non-deterministic behavioral specification, both in timing and in functions. Questions involved other approaches to formalizing VHDL (which are, according to Goldschlag, operational) and the advisability of adding features to VHDL. Goldschlag responded that his intent was not to affect the language, but to explore VHDL as an interesting programming language in its own right.

The next paper was “Experiences Formally Verifying a Network Component” by Paul Curzon (University of Cambridge), reporting on the verification of a small component of a network. This is a real, fabricated component that is in use, but was designed with no thought for formal verification. Curzon gave a summary of the application, which is a packet (communications) switch, and discussed the seven-week verification process.

## 11. Safety II

“Evaluating Software for Safety Systems in Nuclear Power Plants” by J. Dennis Lawrence, Warren L. Persons, and G. Gary Preckshot (Lawrence Livermore National Laboratory), and John Gallagher (U.S. Nuclear Regulatory Commission [NRC]) described some of the work done by the NRC in investigating methods for evaluating software in nuclear power plants. The NRC conducted a workshop with technical experts and investigated practices used by industry in developing safety-critical software.

Amer Saeed, Rogerio de Lemos, and Tom Anderson (University of Newcastle) presented “An Approach for the Risk Analysis of Safety Specifications.” This talk dealt with the risk analysis of the results of the requirements phase for software. The aim is to locate and remove faults introduced in the requirements phase. The methodology for risk analysis focuses on the analysis of the safety requirements. It consists of a framework with phases of analysis, a graph that depicts the relationship between the safety specifications, a set of formal techniques for the issues to be analyzed, and a set of procedures for the risk analysis of the safety specifications.

“Causality as a Means for the Expression of Requirements for Safety Critical Systems” was presented by Andrew Coombes, John McDermid, and Philip Morris (University of York). This talk described a method for the development of requirements for software, in particular, software for safety-critical applications. The method described uses formal methods as the underlying principal and involves modeling three main components: the environment into which the system is embedded; the fundamental requirements or system goals; and the derived requirements, which result by considering how to satisfy the fundamental requirements in the specific environment. An example was given of modeling the fuel management system for a fighter aircraft. The modeling technique is described as “work in progress,” with more research needed. Future work includes developing tools, performing case studies, developing concrete syntax and semantics, and using causal logic to animate the specifications.

## 12. Security

“Covert Channels Here to Stay?” by Ira S. Moskowitz and Myong H. Kang (NRL) covered a new metric, the small message criterion, for use in the analysis of reducing the threat of covert channels without crippling performance.

Charles N. Payne, Andrew P. Moore, and David M. Mihelcic (Naval Research Laboratory [NRL]) submitted “An Experience Modeling Critical Requirements” which discussed NRL’s experience and lessons learned in designing a Selective Bypass Device (SBD) application.

“On Measurement of Operational Security” by Sarah Brocklehurst and Bev Littlewood (City University), and Tomas Olovsson and Erland Jonsson (Chalmers University of Technology) covered the results of an experiment in operation security using college students to break into a computer system.

## 13. Evening Event

The COMPASS ’94 banquet speaker, Professor Brian Randell of the University of Newcastle upon Tyne, summed up COMPASS this way:

COMPASS is filling a need that no other conference is attempting. COMPASS recognizes that problems in security may be shared by software safety and system safety, and vice versa. In both cases, reliability is the goal to be achieved. COMPASS looks at formal proofs, testing, and fault tolerance methods as complementary instead of rival approaches. Most of all, COMPASS is bringing together software and hardware communities, and security and safety communities from industry, government and academia.

Together, these communities may make great gains in solving the problems of providing computer assurance in complex systems, such as aerospace systems, medical devices, military weapons, and transportation.

## 14. COMPASS ’95

COMPASS ’95 will be held June 26–30, 1995, at NIST in Gaithersburg, Maryland. The deadline for papers submitted for COMPASS ’95 is January 14, 1995. For information about COMPASS ’95 or how to obtain proceedings of COMPASS ’94, contact Dolores Wallace, Computer Systems Laboratory, National Institute of Standards and Technology, Building 225, Room B266, Gaithersburg, MD 20899-0001; telephone (301) 975-3340 or fax (301) 926-3696.

